# The State of Telepathology in Africa in the Age of Digital Pathology Advancements: A Bibliometric Analysis and Literature Review

**DOI:** 10.7759/cureus.63835

**Published:** 2024-07-04

**Authors:** Mohammed El Jiar, Imane Eliahiai, Sanae Chaib, Khalid Elmorabit, Mohamed Mouatakid, Jinane Kharmoum, Mariame Chraibi

**Affiliations:** 1 Faculty of Medicine and Pharmacy of Tangier, Abdelmalek Essaadi University, Tangier, MAR; 2 Department of Pathology, University Hospital Mohammed VI of Tangier, Tangier, MAR

**Keywords:** telemedicine, pathology, bibliometric study, healthcare systems, africa, telepathology

## Abstract

Telepathology emerges as a vital tool, offering significant promise for enhancing pathology services in Africa, a region historically challenged by healthcare access and resource limitations. This review explores the development, adoption, and impacts of telepathology in Africa through a comprehensive bibliometric analysis and literature review. A methodical search in PubMed for publications up to 2024 revealed 119 pertinent studies, out of which 47 met the inclusion criteria for a focused review on telepathology's role in African healthcare settings. This research has charted a clear trajectory of growing interest in telepathology, as evidenced by the annual increase in related publications and robust international collaboration. It underscores the expanding utility of telepathology in diagnostics, education, and research within Africa, particularly in domains like dermatopathology, neuropathology, and, notably, oncology. The integration of artificial intelligence into telepathology presents new frontiers for enhancing diagnostic accuracy and efficiency. However, the review also identifies persistent challenges such as infrastructural inadequacies, a shortage of skilled professionals, and regulatory hurdles. The study highlights the indispensable role of international partnerships in advancing telepathology in the region. This review proposes a strategic pivot toward "leapfrogging," an approach that allows Africa to skip traditional developmental hurdles by directly adopting cutting-edge technologies and practices.

## Introduction and background

Telepathology, a significant branch of telemedicine, has emerged as a pivotal innovation, enabling the exchange and interpretation of pathological data over distance through telecommunications technology [1]. This discipline, which is integral to the expanding domain of digital pathology, facilitates remote diagnostics, thereby dismantling geographical barriers that previously hindered the accessibility and efficiency of pathology services. Defined as the practice of pathology at a distance using telecommunication means, telepathology was conceptualized in the early 1980s, marking a transformative era in medical diagnostics [2]. Its evolution traces back to the pioneering application of telecommunication for transmitting static images, progressing towards dynamic telepathology with real-time video feeds, and culminating in the advent of whole slide imaging (WSI) [3]. These milestones reflect a trajectory of technological and methodological advancements aimed at enhancing the fidelity and utility of remote pathology consultations.

Telepathology systems are broadly categorized into static, dynamic, and virtual slide systems [4]. Static telepathology involves the transmission of pre-captured digital images, suitable for cases where real-time interaction is not critical. Conversely, dynamic telepathology enables live, interactive analysis through video microscopy, necessitating a robust bandwidth for real-time data transmission. Virtual slide systems represent a synthesis of the static and dynamic modalities, leveraging WSI to produce high-resolution, digital facsimiles of entire slides. Each modality addresses distinct operational and diagnostic needs, ranging from primary diagnoses and inter-institutional consultations to educational purposes and quality control measures.

The integration of telepathology into healthcare systems has been catalyzed by significant advancements in digital imaging, artificial intelligence (AI), and telecommunications [5]. These technological strides have not only expanded the capabilities and reliability of telepathology services but have also streamlined the diagnostic process, enhancing the speed and accuracy of pathological assessments [6]. Furthermore, the incorporation of AI algorithms offers the promise of automated preliminary analyses, potentially revolutionizing diagnostic workflows and accuracy [7].

Despite the clear benefits and transformative potential of telepathology, its adoption and implementation in Africa are confronted with unique challenges. Amidst these advancements, the African continent faces a unique set of challenges in adopting telepathology, notably underscored by a pronounced shortage of pathologists [8]. Moreover, the continent's vast, unevenly distributed population, compounded by variable telecommunications infrastructure and differing regulatory contexts, presents a complex matrix of barriers to widespread telepathology deployment [9]. However, these challenges coexist with unparalleled opportunities to leapfrog conventional healthcare delivery limitations, offering a pathway to significantly improve access to specialized diagnostic services.

This paper presents a comprehensive overview and bibliometric analysis of the application and development of telepathology in the African healthcare context, with a specific focus on its implication amid digital pathology advancements.

## Review

Methodology

We conducted a comprehensive search using the PubMed database to seek documents published up to 2024. The search string utilized included keywords related to telepathology and Africa with the list of all African countries: (telepathology OR "remote pathology" OR "digital pathology" OR "whole slide imaging" OR "virtual microscopy") AND (Africa OR Algeria OR Angola ... OR Zambia OR Zimbabwe).

A bibliometric study is a valuable method for determining the overall trend of research activity and elucidating the connections between relevant research institutions [10]. Bibliometrics provide a valuable means of assessing the evolution of scientific publications across different countries and periods in various fields and are particularly useful for emerging disciplines including biomedical fields [11]. However, to date, no bibliometric analyses have been published to illustrate the trends in telepathology applications in African healthcare settings. This study provides an analysis of the related published data using VOSviewer (Centre for Science and Technology Studies, Leiden, The Netherlands) and Bibliometrix tools [12]. This step involved examining publication metrics, co-authorship networks, and keyword occurrences to identify research trends, influential authors, and collaborative networks within the scope of telepathology in Africa.

Records retrieved in the initial search underwent a selection process using the Rayyan tool (Qatar Computing Research Institute, Doha, Qatar) to identify relevant studies to be included in the analysis [13]. We included articles in English, French, and German that reported on telepathology healthcare systems in African countries or studies conducted in African countries that involved aspects of telepathology, such as digital pathology. Exclusions were made for general reviews and articles that broadly approached telepathology without a specific focus on African implementations.

**Figure 1:**
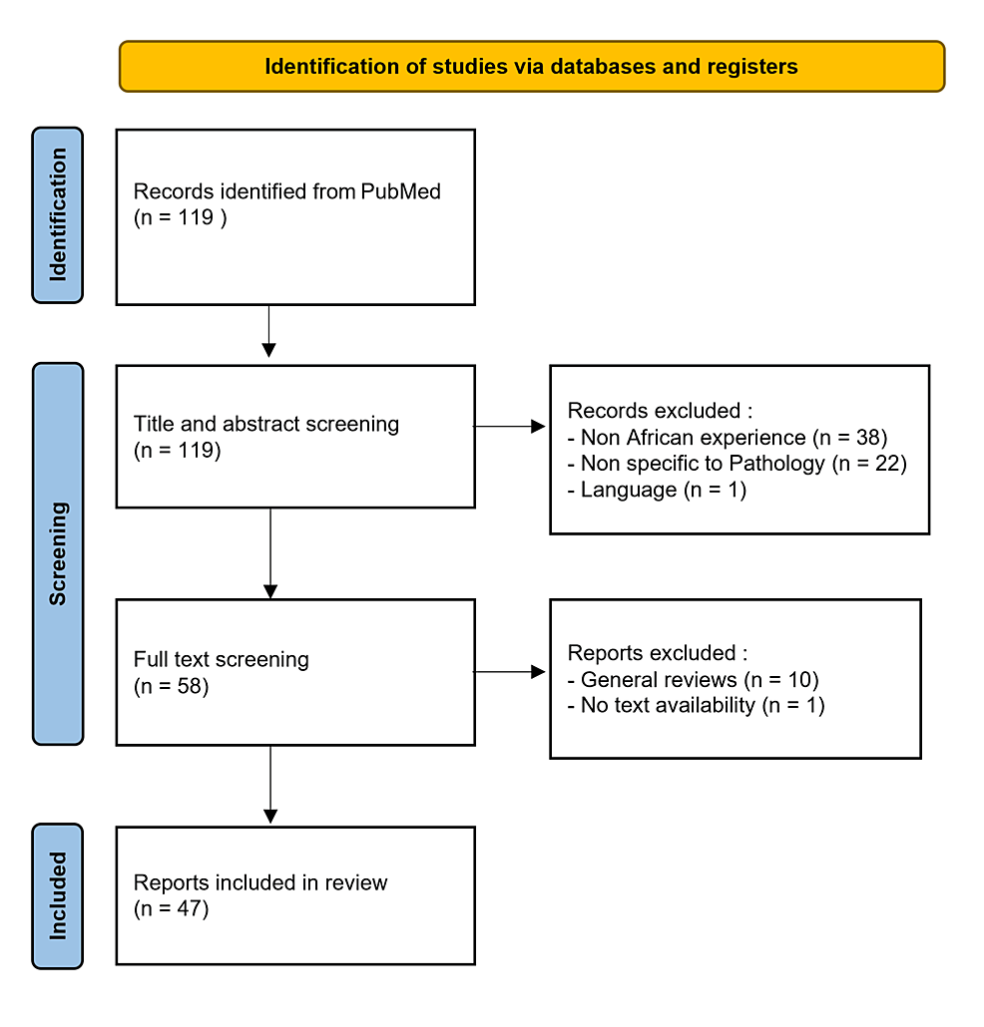
Flow diagram of publication selection for telepathology research in Africa from PubMed.

Two authors independently screened titles and abstracts against the inclusion criteria. Discrepancies were resolved through discussion or consultation with a third researcher. The process of screening titles and abstracts led to the exclusion of 61 articles, and after a detailed full-text review of the remaining 58, 47 publications were selected based on predefined inclusion criteria (Figure 1).

Two team members independently extracted data using Google sheets. Extracted data included the publication year, author(s), title, a summary of the telepathology experience reported, application field, presence of international collaboration, and outcomes, including any noted evolution or future perspectives (Table 1).

**Table 1:** Publications included that reports a telepathology experience in an African countries.

Authors	Title	Year	Country	Field of use	Technology	Collaborator	Findings
Gulube and Wynchank [14]	Telemedicine in South Africa: Success or Failure?	2001	South Africa	Diagnosis	Unspecified	None	South Africa initiated its first national telemedicine program in 1998, covering teleradiology, tele-ultrasound for antenatal services, telepathology, and tele-ophthalmology across 28 sites. Implementation faced challenges and delays, and the outcomes of telepathology were not reported.
Ayad and Sicurello [15]	Telepathology in Emerging Countries Pilot Project Between Italy and Egypt.	2008	Egypt	Teleconsultation	Static, dynamic	Italy	The first telepathology project at the Italian Hospital in Cairo, in collaboration with Civico Hospital in Palermo, yielded positive outcomes in diagnosing difficult cases, saving time and money, and enhancing education, although no metric evidence was provided.
Banach et al. [16]	Dynamic Active Telepathology over National Health Laboratory Service Network, South Africa: Feasibility Study Using Nikon Coolscope	2008	South Africa	Teleconsultation	Dynamic	None	Digital dynamic pathology systems were deployed in Mthatha, East London, and Port Elizabeth, South Africa, to mitigate pathologist shortages. Connected through LAN or WAN, these systems facilitated 60 review sessions in Cape Town and Pretoria, showing particular benefits in dermatopathology, malignant neoplasia, and hematopathology, with LAN offering a superior experience.
Wamala et al. [17]	Feasibility and Diagnostic Accuracy of Internet-Based Dynamic Telepathology Between Uganda and Germany	2011	Uganda	Diagnosis	Dynamic, robotic	Germany	The study showed a 97% diagnostic agreement in 96 cases between Mulago Hospital in Uganda and Fuerth Hospital in Germany using telepathology, compared to conventional microscopy.
Ayad [18]	Virtual Telepathology in Egypt Applications of WSI in Cairo University	2011	Egypt	Education, Diagnosis	WSI	Italy	Launched in 2003 and gaining international collaboration by 2004, this project established DTU at Cairo University, significantly advancing digital pathology for education and diagnostics, showing success in challenging case consultations and expanding into postgraduate education.
Pagni et al. [19]	Virtual Surgical Pathology in Underdeveloped Countries: The Zambia Project	2011	Zambia	Diagnosis	WSI	Italy	The study details a Zambian telepathology project aimed at enhancing diagnostic accuracy and access, demonstrating a high agreement with traditional microscopy and telepathology's promise in underdeveloped regions.
Patel and Douglas [20]	Creating a Virtual Slide Map of Sputum Smears by Auto-stitching	2011	South Africa	Research	Static	None	The study highlighted a technique for enhancing tuberculosis screening by scanning sputum smears, and auto-stitching them into a virtual slide map, thereby speeding up diagnosis and reducing technician dependency. It confirmed that geometric hashing and SIFT methods are effective for creating virtual slides and supporting TB diagnosis and research.
Sohani and Sohani [21]	Static Digital Telepathology: A Model for Diagnostic and Educational Support to Pathologists in the Developing World	2012	Kenya, Tanzania	Diagnostic, Education	Static	International	The project aimed to address challenges such as equipment costs, maintenance, and internet connectivity in developing countries by introducing static imaging systems as a simpler, cost-effective solution. It involved donating equipment, training local pathologists, and uploading images to telepathology servers for consultation.
Ayad and Yagi [22]	Virtual Microscopy Beyond the Pyramids: Applications of WSI in Cairo University for E-Education & Telepathology	2012	Egypt	Education and teleconsultation	WSI	Italy, the UK, the United States	The project introduced static imaging systems in developing countries to tackle high costs, maintenance, and connectivity issues, involving equipment donation, pathologist training, and image-based consultations. Over 40 months, 109 cases were reviewed, with static images allowing for complete or partial diagnosis in 91.7% of them, proving static digital telepathology as a cost-effective, reliable solution for improving diagnostic and educational services.
Gimbel et al. [23]	A Static-Image Telepathology System for Dermatopathology Consultation in East Africa: The Massachusetts General Hospital Experience	2012	Kenya, Tanzania	Dermatopathology	Static	United States	The study assessed a telepathology platform linking four African hospitals with Massachusetts General Hospital for dermatopathology consultations, addressing pathologist shortages. It reported 91% diagnostic accuracy and high interobserver agreement (K = 0.86), proving the approach's effectiveness in resource-limited environments.
Kumar et al. [24]	Telecytology in East Africa: A Feasibility Study of Forty Cases Using a Static Imaging System	2012	Kenya, Tanzania	Cytology	Static	United States	The pilot study of forty challenging cytology cases from East Africa, analyzed by six pathologists via static images on a telepathology site, aimed to assess telecytology's utility in remote diagnostics. With diagnostic concordance between 71% and 93% and consensus matching histological diagnoses when biopsies were done, the study acknowledged issues like image quality but validated telecytology's feasibility in East Africa.
Fischer et al. [25]	Establishing Telepathology in Africa: Lessons from Botswana	2013	Botswana	Diagnosis, Education	Robotic	Unspecified	The article reviews telepathology in Botswana, noting the Zeiss Mirax Live system's deployment, and challenges like processing, connectivity, and power, underscoring the importance of government backing and adaptation to local needs, proving its effectiveness in improving healthcare and education in resource-limited areas.
Perron et al. [26]	Online Teaching of Inflammatory Skin Pathology by a French-Speaking International University Network	2014	France, Québec, Switzerland, Ivory Coast	Dermatopathology, Education	WSI	France	Launched in 2011, a French university network developed online dermatopathology education modules, expanding internationally with virtual slides and cases. The project successfully enhanced the inflammatory skin pathology module, demonstrating online education's potential.
Micheletti et al. [27]	Robotic Teledermatopathology from an African Dermatology Clinic	2014	Botswana	Dermatopathology	Robotic	United States	Botswana's implementation of robotic telepathology significantly enhanced the diagnosis and treatment of complex dermatologic cases, often changing initial diagnoses and improving patient care by identifying conditions that local diagnostics missed.
Mpunga et al. [28]	Diagnosis of Cancer in Rural Rwanda: Early Outcomes of a Phased Approach to Implement Anatomic Pathology Services in Resource-Limited Settings	2014	Rwanda	Diagnosis	Static	United States	In rural Rwanda, Butaro District Hospital's staged pathology service setup, covering equipment purchase, infrastructure upgrade, and staff training, enhanced local cancer diagnosis with 437 specimens processed in six months of 2012. This proved the viability of pathology services in resource-scarce areas, achieving a median reporting time of 32 days.
Carey et al. [29]	Remote and Rapid Pathological Diagnosis in a Resource-Challenged Unit	2014	Malawi	Diagnosis, Pediatric Oncology	Static	None	Malawi's pediatric oncology unit adopted a telepathology system with static imaging to speed up and enhance diagnostic accuracy, positively affecting patient care.
Bowa and Anderson [30]	Using Virtual Microscopy at Copperbelt University	2014	Zambia	Education	WSI	None	The implementation of virtual microscopy involved digital slide acquisition and training for staff and students, modernizing medical education. It notably enhanced resource accessibility, student engagement, and hinted at digital pathology's diagnostic potential.
Mpunga et al. [31]	Implementation and Validation of Telepathology Triage at Cancer Referral Center in Rural Rwanda	2016	Rwanda	Diagnosis	Static	United States	The study details establishing a telepathology system to enhance cancer care, from training and setting up internet connections to using online tools for diagnosis. It achieved over 95% diagnostic agreement with traditional methods, proving its efficacy in improving accuracy and capacity in resource-limited settings.
Streicher et al. [32]	Innovative Dermatopathology Teaching in a Resource-Limited Environment	2016	Ethiopia	Dermatopathology, Education	Static	None	A 3-week dermatopathology course used an iPhone-microscope slide projection system to tackle resource and training shortages, significantly enhancing resident knowledge and satisfaction.
Rotimi et al. [33]	Remote Teaching of Histopathology Using Scanned Slides via Skype Between the United Kingdom and Nigeria	2017	Nigeria	Education	WSI	UK	In Nigeria, consultant pathologists and trainees used digital slides and Skype for interactive lectures, improving pathology education in resource-scarce areas. Participants reported high satisfaction with the system's ease of use and the educational value for local practice.
Royall et al. [34]	From Access to Collaboration: Four African Pathologists Profile Their Use of the Internet and Social Media	2017	Nigeria, Uganda, Tanzania	Teleconsultation, Education	Static	Sub-Saharan Africa	The article highlights how African pathologists use the internet and social media to enhance pathology services in SSA, sharing four pathologists' experiences. It notes improvements in diagnostics, faster results, and better collaboration, with challenges like costs, power reliability, and the need for dependable information sources.
Voelker et al. [35]	Diagnostic Validity of Static Telepathology Supporting Hospitals Without Local Pathologists in Low-income Countries	2018	Germany, Tanzania	Diagnosis	Static	Germany	The study details telepathology (TP) setup and training in a hospital lacking a resident pathologist, reporting on 545 cases. Seventy percent of TP diagnoses were confirmed by second opinions, with an 84% overall diagnostic usefulness. Minor deviations occurred in 14% of cases, while 5% were misinterpreted benign/malignant, and 8% were insufficient for TP diagnosis, indicating specific disease subgroups had higher accuracy.
Montgomery et al. [36]	Practical Successes in Telepathology Experiences in Africa	2018	Malawi	Diagnosis	WSI	United States	The article describes telepathology at Kamuzu Central Hospital in Malawi, emphasizing improved diagnostics and education through collaboration between local and US pathologists. It highlights the service's role in enhancing patient care, aiding clinical trials, and offering weekly educational conferences, thus supporting quality improvements.
Ayad et al. [37]	Ki 67 Assessment in Breast Cancer in an Egyptian Population: A Comparative Study Between Manual Assessment on Optical Microscopy and Digital Quantitative Assessment	2018	Egypt	Research, AI	WSI	Unspecified	The study shows digital pathology can standardize Ki-67 scoring in breast cancer, finding good agreement between manual and automated methods but noting manual scores often overestimate compared to automated, especially with different high Ki-67 cutoffs.
Völker et al. [38]	Ten Years of Telepathology for a Mission Hospital in Tanzania	2019	Tanzania	Diagnosis, Teleconsultation	Static	Germany	Tanzania's telepathology with iPath reduced diagnosis times from weeks to days, examining 5,230 cases over ten years, mainly identifying common tumors and infections rather than exotic diseases.
Voelker et al. [39]	Re-evaluation of Challenging Telepathological Diagnoses in Support of a Hospital in Tanzania	2019	Tanzania, Germany	Diagnosis	Static	Germany	The study re-evaluated challenging telepathology cases, finding discrepancies across six levels, from identical to undiagnosable. For benign cases, 62% matched original diagnoses; for malignant, 42%. Despite limitations, telepathology significantly aids pathology services in low-income countries.
Martines et al. [40]	Pathology and Telepathology Methods in the Child Health and Mortality Prevention Surveillance Network	2019	Haiti, Rwanda	Diagnosis, Child Mortality	WSI, Static	International	The document introduces standardized checklists and algorithms for histopathologic findings and infectious disease testing in MITS, using telepathology to enhance collaboration and pathology capacity in CHAMPS project countries, ultimately aiming to inform interventions for child mortality.
Trabelsi et al. [41]	An Immunoscore System Based On CD3+ And CD8+ Infiltrating Lymphocytes Densities To Predict The Outcome Of Patients With Colorectal Adenocarcinoma	2019	Tunisia	Research	WSI	Unspecified	The study aimed to validate the Immunoscore (IS) in colorectal adenocarcinoma, comparing it with TNM staging and assessing its predictive value for adjuvant treatment. IS showed significant correlation with 5-year overall and disease-free survival, surpassing TNM's predictive ability, especially for survival analysis, indicating better outcomes with high IS.
Voelker et al. [42]	Telepathological Evaluation of Paediatric Histological Specimens in Support of a Hospital in Tanzania	2020	Germany, Tanzania	Pediatric , Diagnosis, Teleconsultation	Static	Germany	The study examines telepathology's role in pediatric diagnostics in resource-limited settings, noting prevalent diagnoses and the need for secondary evaluation in Germany for challenging cases. While achieving 70% concordance for benign diseases, it highlights limitations and suggests additional diagnostic methods.
Zerd et al. [43]	Photomicrograph-Based Neuropathology Consultation in Tanzania: The Utility of Static-Image Teleneuropathology in Low- and Middle-Income Countries	2020	Tanzania	Neuropathology, Teleconsulting	Static	United States	In Tanzania, a study reviewed 75 neuropathologic cases using static-image teleneuropathology to enhance diagnostic accuracy. It achieved a 71% agreement with on-site glass diagnosis under strict criteria and 88% with less stringent criteria, signifying a notable improvement from the initial 36% by general pathologists to 71% with static telepathology.
Santos et al. [44]	Highlights from the 4th PALOP-AORTIC Conference on Cancer, 29–31 July 2020, Luanda, Angola	2020	Mozambique, Angola	Teleconsultation, Education	Static, WSI	Spain, United States	The report summarizes the 4th PALOP-AORTIC cancer conference, highlighting efforts to control cancer in Portuguese-speaking African countries. It emphasizes education, telepathology, and collaborative research to tackle the increasing cancer burden in PALOP. Specific outcomes include proposals for the PALOP oncology group and discussions on cancer control strategies and training programs.
Ayad et al. [45]	Immunohistochemical Study of Ezrin Expression in Colorectal Carcinoma: A Comparative Study between Objective Method and Digital Quantitative Assessment	2020	Egypt	Research	WSI	Unspecified	The study compared visual and digital assessment of Ezrin expression in colorectal cancer tissues, finding significantly higher Ezrin levels in cancerous tissues compared to adjacent normal mucosa. Ezrin was expressed in 92.2% of CRC cases, correlating with tumor grade. Results showed a strong correlation (R = 0.868) between subjective and quantitative methods, suggesting digital pathology enhances diagnostic accuracy and reliability.
Azakpa et al. [46]	Telepathology Practice in Cancer Diagnosis in Saint Jean de Dieu Hospital - Tanguieta Benin	2021	Benin	Teleconsultation	WSI	International	Implemented telepathology at Saint Jean de Dieu Hospital, Benin, training local technicians by European pathologists. Analyzed 1593 patients' specimens (2016-2018), resulting in 399 cancer diagnoses. Improved cancer diagnosis, notably in breast and prostate cancer grading.
Gruber-Mösenbacher et al. [47]	Digital Pathology in Cameroon	2021	Cameroon	Diagnosis	Static, WSI	International	Leveraged digital pathology to address pathologist shortages, transitioning from mobile phone imagery to whole slide imaging. Improved diagnostic accuracy and efficiency, enhancing cancer treatment planning and overcoming geographic barriers.
Benson et al. [48]	Use of Telepathology to Facilitate COVID-19 Research and Education through an Online COVID-19 Autopsy Biorepository	2021	United States, South Africa	Education, Research	WSI	United States	Established an online COVID-19 autopsy histology biorepository with high-resolution digital slides to support remote research and education. Utilized telepathology to overcome pandemic-related limitations on traditional autopsy and pathology studies, facilitating multiple research projects and educational conferences.
Mremi et al. [49]	The Role of Telepathology in Diagnosis of Pre-malignant and Malignant Cervical Lesions: Implementation at a Tertiary Hospital in Northern Tanzania	2022	Tanzania	Diagnosis, Cervical lesions	WSI	Denmark	Study shows 87.7% overall concordance between conventional microscopy and scanned images by three pathologists, suggesting telepathology's accuracy for primary surgical pathology diagnosis. Intra- and inter-observer agreement high for cervical lesions, with discrepancies primarily in pre-malignant lesion interpretation.
Kasonkanji et al. [50]	Clinical Characteristics and Outcomes of Acute Lymphoblastic Leukemia in Adolescents and Young Adults in Malawi	2022	Malawi	Teleconsultation, Diagnosis, Lymphoma	Unspecified, Dynamic	United States	Observational cohort study with 19 participants provides insights into baseline characteristics, treatment modalities, and outcomes. 11 out of 15 patients achieved remission after treatment initiation, but 10 eventually relapsed. 12- and 24-month overall survival rates were 50% and 17%, respectively, with worse survival associated with CNS involvement.
Silas et al. [51]	Telepathology in Nigeria for Global Health Collaboration	2022	Nigeria	Diagnosis, Teleconsultation	Static, WSI, Dynamic, Robotic	United States	The document emphasizes the critical need for telepathology in Nigeria due to pathology personnel shortage and setup challenges. While specific outcomes are not detailed, telepathology offers broad benefits including quality assurance, cost reduction, faster pathology reports, and improved capacity for research and teaching.
El Agouri et al. [52]	Assessment of Deep Learning Algorithms to Predict Histopathological Diagnosis of Breast Cancer: First Moroccan Prospective Study on a Private Dataset	2022	MOROCCO	AI, Research	WSI	Unspecified	Researchers use ResNet50 and Xception deep learning models on their dataset for image classification. Focus is on categorizing images into normal tissue, benign lesions, in situ carcinoma, and invasive carcinoma. Xception slightly outperforms ResNet50, achieving 88% overall accuracy and 95% sensitivity for carcinoma detection, highlighting deep learning's potential in breast cancer diagnosis.
McAlpine et al. [53]	The Dynamics of Pathology Dataset Creation Using Urine Cytology as an Example	2022	South Africa	AI, Research	WSI	Unspecified	The article describes creating a pathology dataset, involving image labeling by pathologist consensus, recording labeling dynamics, and implementing quality assurance measures. It highlights time and resource requirements, variations in labeling time across diagnostic categories, and challenges in reproducibility of the atypical urothelial category within The Paris System.
McAlpine et al. [54]	Is It Real or Not? Toward Artificial Intelligence-based Realistic Synthetic Cytology Image Generation to Augment Teaching and Quality Assurance in Pathology	2022	South Africa	AI, Education	WSI	Unspecified	The article details using a StyleGAN3 model to generate synthetic images of malignant urine cytology. It discusses the potential to overcome limitations in teaching materials and addresses technical and morphological challenges. The GAN model successfully produces realistic and diverse images but notes some unrealistic and artifactual images, stressing the need for manual curation in educational contexts.
Vassilakos et al. [55]	Telecytologic Diagnosis of Cervical Smears for Triage of Self-sampled Human Papillomavirus–Positive Women in a Resource-limited Setting: Concept Development Before Implementation	2023	Cameroon	Cervical Lesions, Cytology	WSI	Switzerland	The study validated manual preparation and digitization of cervical smears, showing it as a feasible method. Diagnostic accuracy was compared between virtual and traditional microscopy in detecting cervical intraepithelial neoplasia grade 2 or worse in 264 HPV-positive Cameroonian women. Screening digital slides performed comparably to glass slides, suggesting telecytologic diagnosis using digital slides could effectively triage HPV-positive women.
Mremi et al. [56]	Diagnostic Validation of a Portable Whole Slide Imaging Scanner for Lymphoma Diagnosis in Resource-constrained Setting: A Cross-sectional Study	2023	Tanzania, Uganda	Lymphoma, Diagnosis	WSI	UK	The study evaluated a mobile WSI device by comparing its diagnostic concordance with GSM in lymph node biopsy specimens suspected of lymphoma. It assessed morphological features in both modalities and analyzed diagnostic agreement using Cohen's kappa coefficient. Overall diagnostic concordance for lymphoma, metastatic, and benign conditions was high, with WSI demonstrating sensitivity and specificity of 95.2% and 85.7% for lymphoma detection, respectively. Inter-observer agreement was high at 0.86.
Kothari et al. [57]	Increasing Access to Pathology Services in Low- and Middle-Income Countries Through Innovative Use of Telepathology	2023	Uganda	Diagnosis	Robotic	United States	A study in Uganda implemented telepathology at a surgery center, examining tissue samples remotely. Among 110 patients from April 2021 to July 2022, common malignancies included esophageal squamous cell carcinoma, ductal breast carcinoma, and colorectal adenocarcinoma.
Hasan et al. [58]	Clinico-Pathological Features and Immunohistochemical Comparison of p16, p53, and Ki-67 Expression in Muscle-Invasive and Non-Muscle-Invasive Conventional Urothelial Bladder Carcinoma	2023	Egypt	Research	WSI	Unspecified	The study classified 62 bladder urothelial cancer cases into muscle-invasive (MIBC) and non-muscle-invasive (NMIBC) groups. Results showed no significant difference in p53 expression between MIBC and NMIBC using a 20% cutoff. Ki-67 expression correlated with higher grade and muscle invasion, while negative p16 immunostaining was linked to lower grade and NMIBC, especially in papillary pattern cases.
Manirakiza et al. [59]	The Use of Vsee Videoconferencing for Live Telepathology in Rwanda: A Potential Solution for Resource-limited Area	2023	Rwanda	Diagnosis	Robotic	None	In Rwanda, a low-cost telepathology system using Vsee videoconferencing transmitted live histologic images for diagnostic purposes. It achieved substantial agreement with conventional microscopy-based diagnoses, with a Cohen’s kappa of 0.77 and 76.6% perfect agreement for 60 cases.
McAlpine et al. [60]	Are Synthetic Cytology Images Ready for Prime Time? A Comparative Assessment of Real and Synthetic Urine Cytology Images	2023	South Africa	Research	WSI	Unspecified	Synthetic urine cytology images were generated and compared with real images through an online survey filled out by pathology personnel. Results showed no significant difference in diagnostic error rates or subjective image quality scores between real and synthetic images. Both types were equally likely to be selected for inclusion in teaching sets.

Results

Bibliometric Analysis

The bibliometric analysis identified a total of 119 articles across 71 journals. The very first publication dates back to 2001. This research has demonstrated a notable annual growth rate of 6.21%, contributed by 953 authors with an average collaboration of 10.6 co-authors per document. The analysis highlighted a robust level of international collaboration, accounting for 46.22% of the publications.

**Figure 2:**
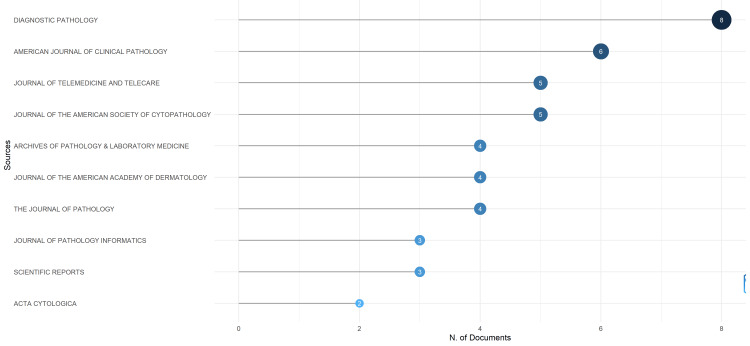
Distribution of top 10 journals publishing telepathology research on Africa from 2001 to 2023.

**Figure 3:**
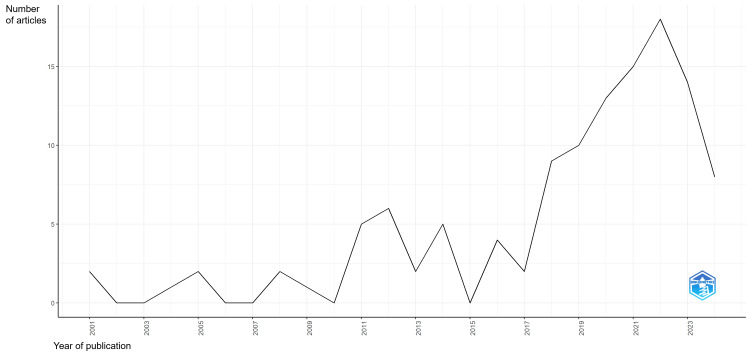
Annual scientific production in telepathology research publications focused on Africa.

Source and publication trends : Among these sources, *Diagnostic Pathology*, the *American Journal of Clinical Pathology*, the *Journal of Telemedicine and Telecare*, and the *Journal of the American Society of Cytopathology* emerged as the predominant journals for disseminating telepathology findings (Figure 2). A significant increase in publications was observed between 2020 and 2023, peaking in 2022 with 18 articles (Figure 3).

**Figure 4:**
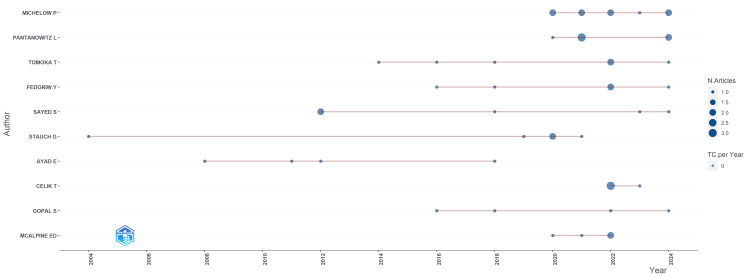
Evolution of author contributions in African telepathology research over time. N.Articles, number of articles; TC per Year, total citations per year

Key contributors: The analysis identified Michelow, Pantanowitz, and Tomoka as the most prolific authors, highlighting their substantial contributions to the field (Figure 4). Leading affiliations included globally recognized institutions such as the University of Modena and Reggio Emilia and Brigham and Women's Hospital, alongside key African institutions like the University of Rwanda and Butaro District Hospital.

**Figure 5:**
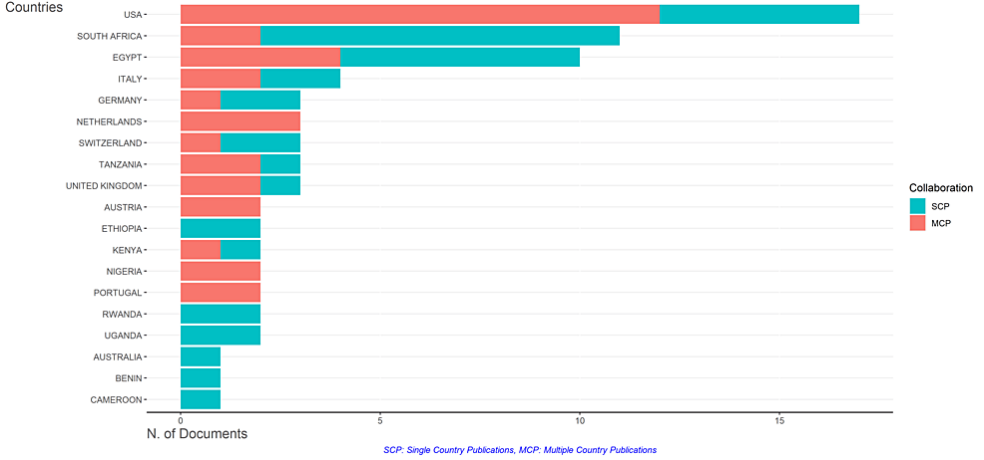
Corresponding author’s countries.

**Figure 6:**
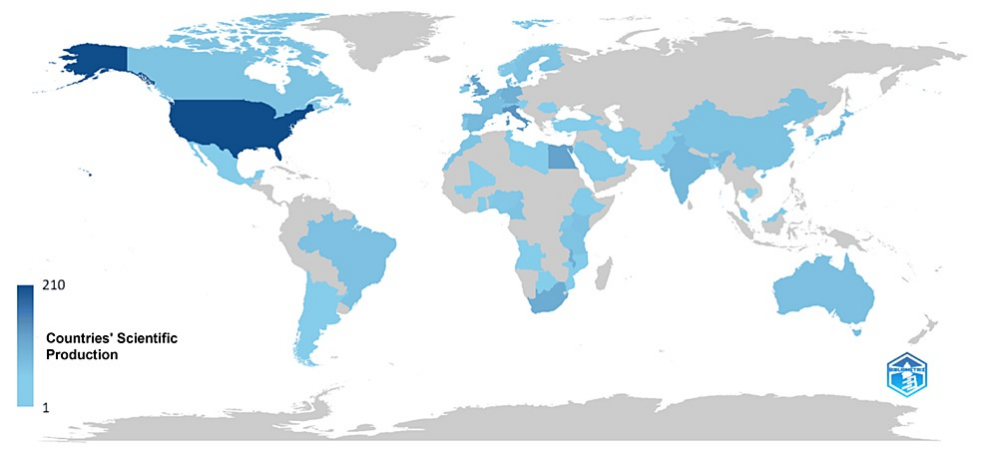
Geographic distribution of telepathology research in Africa.

Geographic distribution : The geographic analysis of the corresponding authors showed the United States, South Africa, and Egypt as leading countries in telepathology research related to Africa (Figures 5-6). Following these were Italy, Germany, the Netherlands, Switzerland, and Tanzania, showcasing the international collaboration and interest in African telepathology. Specifically within Africa, South Africa and Egypt stood out for their research contributions.

**Figure 7:**
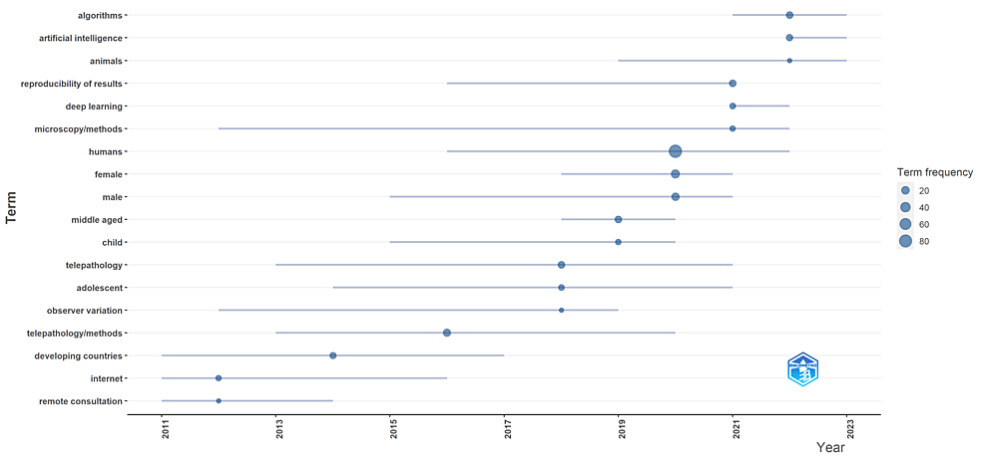
Evolution of trend topics over time on telepathology research in Africa.

**Figure 8:**
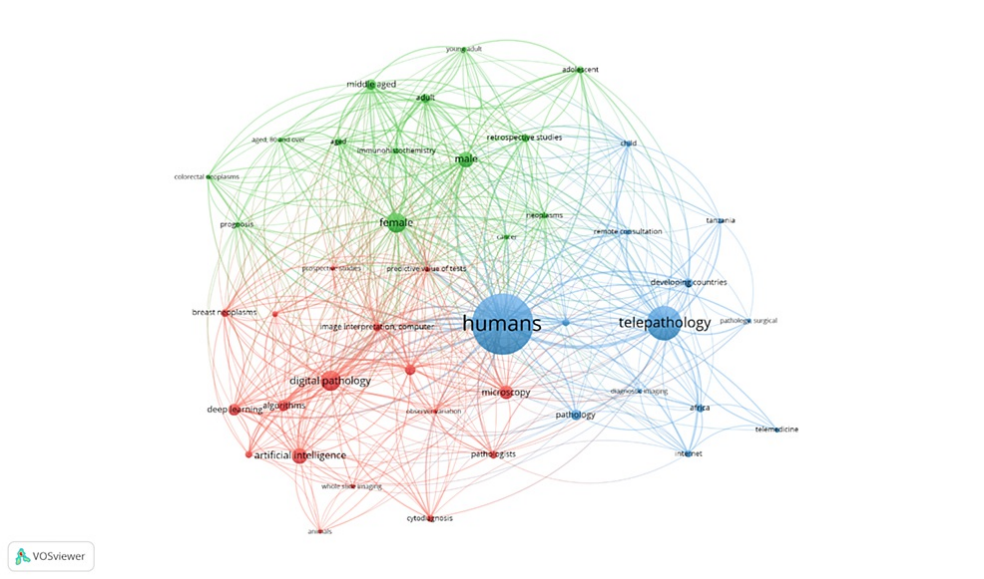
Overlay visualization of co-occurrence of keywords.

Thematic evolution and collaborations: Keyword trends revealed a focus on "telepathology/methods," "algorithms," and "reproducibility of results," with a notable pivot towards "artificial intelligence" in recent years. The transition from early themes of "remote consultation" and "internet" usage in developing countries to advanced topics such as AI since 2022 marks a significant evolution in research focus (Figure 7). The bibliometric network highlighted "Humans" as a central node, connecting closely with "telepathology" and "digital pathology," indicative of these being foundational themes. Emerging links to "artificial intelligence" and "developing countries" signal shifting research priorities (Figure 8). Collaboration patterns, particularly between the United States and South Africa (frequency = 8), as well as Italy and Egypt (frequency = 6), reflect the global collaborative efforts underpinning telepathology advancements in Africa.

**Figure 9:**
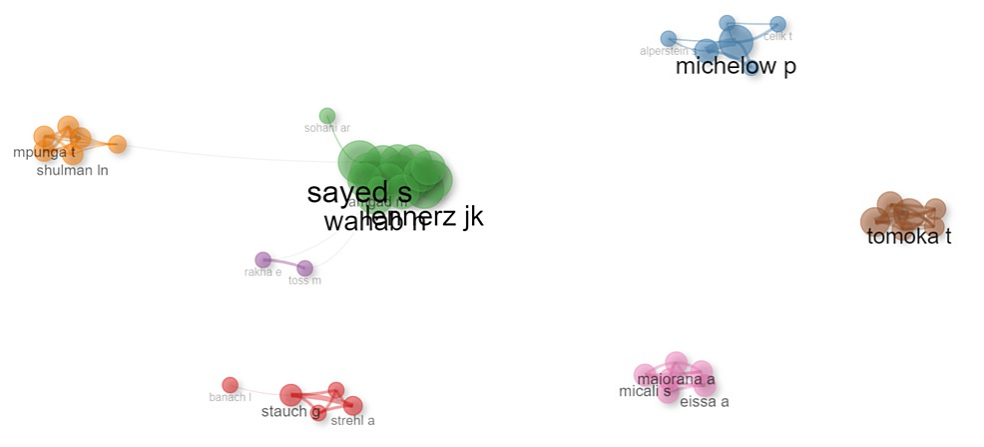
Collaboration network diagram of telepathology researchers.

The collaboration network analysis among telepathology researchers in Africa identified several key contributors based on centrality measures. The top 10 nodes by PageRank are presented in Table 2. The network graph (Figure 9) visually represents these relationships, where node sizes correspond to their centrality measures. "Michelow p" emerged as the most influential node with the highest PageRank of 0.039 and a high betweenness centrality of 4.716, indicating a central role in connecting various subgroups within the network. "Sayed s" also demonstrated significant influence with a PageRank of 0.028 and an exceptionally high betweenness centrality of 125, underscoring its critical function in bridging different clusters. The analysis revealed dense internal connections within clusters. For example, on the network representation, the cluster led by "sayed s" showed substantial collaboration, with notable interactions involving "acosta haab g" and "gingeriadis a" (Figure 9). Similarly, the cluster centered around "stauch g" exhibited strong internal ties, with an important collaboration within this subgroup. 

**Table 2:** Centrality measures of top 10 researchers in the telepathology collaboration network on African context ranked by PageRank.

Node	Cluster	Betweenness	Closeness	PageRank
michelow p	2	4.716	0.2	0.039
stauch g	1	3	0.25	0.029
pantanowitz l	2	1.167	0.167	0.029
sayed s	3	125	0.032	0.028
wahab n	3	44	0.028	0.027
strehl a	1	0	0.2	0.025
grigoriadis a	3	0	0.026	0.025
lennerz jk	3	0	0.026	0.025
salgado r	3	0	0.026	0.025
siziopikou kp	3	0	0.026	0.025

Overview on telepathology use in Africa

The origins of telepathology in Africa trace back to South Africa in 2001, within a broader national telemedicine program that began in 1999 [14]. However, actual projects started in 2008 [15,16]. Among the publications, Tanzania was the most frequently mentioned country, followed by South Africa, Egypt, and then Uganda, Rwanda, and Kenya. The technologies employed varied over time, beginning with static images and progressing to dynamic or robotic microscopes equipped with cameras [17,18]. By 2011, Egypt had integrated WSI, marking a significant advancement in the region's telepathology capabilities [22].

The review revealed a mix of telepathology applications from primary diagnoses and teleconsultations to educational uses, and more recently, engagements in research and the integration of digital pathology technologies. A common theme across many telepathology initiatives was the involvement in international collaborations, particularly with partners from the United States, Italy, and Germany, aligning with the bibliometric analysis that also ranked these countries as primary collaborators.

Chronologically, consistent updates and reports of telepathology experiences were notable in Tanzania, Egypt, and South Africa. This suggests an ongoing engagement with telepathology from as early as 2010 through to the beginning of 2017, highlighting varied and evolving applications mainly in sub-Saharan African countries. Tanzania's telepathology journey was particularly well-documented, providing insight into the structured development of such initiatives within the country. This period also saw other African nations gradually adopting telepathology, indicating a broadening interest and application of this technology across the continent.

Discussion

The number of publications on telepathology in Africa remains comparatively low compared to other bibliometric studies. For instance, Mea conducted a bibliometric study in 2011 using PubMed and identified a total of 967 reports [61]. A more recent study in 2020 by Şenel and Baş identified 1,986 publications on Web of Science [62]. The United States and developed countries continue to be at the forefront of telepathology research, while other regions, such as Africa, are still catching up. This trend is evident in our own findings, despite including African countries in our keyword search. This could be attributed to co-authorship, where African institutions are affiliated with the research but the studies may not directly reflect a true African experience.

The journey of telepathology in Africa commenced with foundational projects aimed at integrating telemedicine systems to address acute healthcare service gaps. In South Africa, the inception of a national telemedicine system in 1998 included telepathology among its services, although it faced implementation challenges that extended the project's timeline without specific outcomes reported for telepathology [13]. This period marked the initial foray into utilizing technology to bridge healthcare delivery gaps in the continent. Subsequently, advancements were made in Egypt, where telepathology projects since the early 2000s demonstrated tangible benefits in diagnostics and education, achieving notable success in challenging case consultations and educational advancements without explicit metrics of success. Similarly, Tanzania's decade-long engagement with telepathology notably reduced diagnostic times from weeks to days, illustrating the technology's potential to significantly enhance healthcare efficiency and access across Africa [38].

Telepathology has experienced significant technological evolution, beginning with static digital systems and progressing to dynamic and robotic telepathology, alongside the integration of WSI and AI. This evolution is evident in the adoption of Nikon Coolscope for dynamic telepathology in South Africa and the implementation of WSI for educational and diagnostic purposes in Egypt and Tanzania [15,17,18]. These advancements have improved the diagnostic accuracy and expanded the scope of telepathology applications.

Subsequent years saw a proliferation of telepathology projects driven by cross-country collaborations, such as the pilot project between Italy and Egypt in 2008, and the dynamic telepathology feasibility study using Nikon Coolscope in South Africa [14,15]. These early collaborations underscored the potential of telepathology for diagnostic, educational, and consultative purposes, even though, they highlighted the necessity for robust infrastructure and reliable internet connectivity. The role of international collaboration and education has been pivotal in the expansion of telepathology in Africa. Projects have leveraged expertise from developed countries and utilized platforms like iPath and Vsee for teleconsultations, as evidenced by Völker et al.'s work in Tanzania and Felix Manirakiza's use of Vsee in Rwanda (2023) [38,58]. These collaborations have not only facilitated rapid diagnostic processes but also fostered educational exchanges, and building local capacities.

The global impact of telepathology is underscored by its ability to bridge the gap in pathology services, particularly in under-resourced regions. Studies from Zambia, Uganda, and Rwanda highlighted the role of telepathology in enhancing diagnostic capabilities and providing educational support [16,18,27,56,58]. The implementation of telepathology in these regions demonstrates a significant improvement in cancer diagnosis, management of infectious diseases, and access to specialized pathology consultations.

The application of telepathology across specific pathologies and specialties, including oncology, infectious diseases, neuropathology and dermatopathology, has shown promising outcomes [19,22,23,25,28,42]. The validation of a portable WSI scanner for lymphoma diagnosis in Tanzania and Uganda and the use of telepathology for cervical lesion diagnosis in Tanzania illustrate its diagnostic accuracy and potential to support cancer care [48,55]. Additionally, the development of an immunoscore system for colorectal adenocarcinoma in Tunisia and the assessment of deep learning algorithms for breast cancer diagnosis in Morocco, leveraging AI and WSI technologies indicates telepathology's expanding role in personalized medicine [40,51]. The establishment of a telepathology system in Benin's Saint Jean de Dieu Hospital significantly improved cancer diagnosis capabilities [45].

Telepathology has also made significant strides in education and research, with initiatives like the online COVID-19 autopsy biorepository showcasing its utility in facilitating research and education despite pandemic-related restrictions [47]. The use of telepathology for training and consultation in Nigeria and the generation of synthetic urine cytology images for educational purposes in South Africa reflect its expanding role beyond diagnostics into educational enhancement and research support [50,59].

Despite its benefits, telepathology faces challenges including infrastructure requirements, data security concerns, and the need for user training. The integration of AI, presents opportunities for enhancing diagnostic accuracy but also necessitates careful validation to ensure reliability.

The main limitation of our study was the inability to conduct a citation analysis using PubMed. This limitation arose due to PubMed's interface and database structure, which primarily focuses on indexing articles but does not provide extensive citation linkage data. Another intrinsic limitation stems from the nature and detail level of the papers included in our review. Our inclusion criteria were designed to broadly identify where telepathology is used and its main outcomes, rather than delving into the depths of detailed telepathology experiences. This approach inherently limited our ability to capture and analyze a common primary outcomes.

## Conclusions

Telepathology in Africa stands as a testament to innovation within the digital pathology landscape, buoyed by the promise of enhanced healthcare access and improved diagnostic accuracy, as revealed through our comprehensive bibliometric analysis and literature review. However, this burgeoning field navigates a complex terrain marked by infrastructure deficiencies, a dearth of local expertise, and regulatory challenges. The reliance on international collaboration, while instrumental in driving initial advancements, underscores a critical dependency that may impede the journey towards a self-sustaining telepathology ecosystem. This review proposes a strategic pivot towards "leapfrogging" - an approach that allows Africa to skip traditional developmental hurdles by directly adopting cutting-edge technologies and practices. By fostering local capacity building and making strategic investments in technology, Africa can leverage telepathology not just to catch up with, but to leap ahead in the global healthcare landscape, transforming challenges into stepping stones towards an autonomous, innovative, and equitable healthcare future.
